# Transmissive hybrid metal–dielectric metasurface bandpass filters for mid-infrared applications

**DOI:** 10.1515/nanoph-2025-0122

**Published:** 2025-10-02

**Authors:** Amr Soliman, Timothy D. Wilkinson

**Affiliations:** Department of Engineering, 2152 University of Cambridge, Cambridge, England

**Keywords:** nanophotonics, hybrid metasurfaces, plasmonics, all-dielectric metasurfaces, mid infrared spectroscopy

## Abstract

Mid-infrared (MIR) spectroscopy plays a pivotal role in molecular identification and biosensing due to its ability to probe characteristic vibrational fingerprints of biomolecules. Plasmonic nanostructures have been explored for MIR applications but suffer from low efficiencies and broad spectral responses caused by intrinsic ohmic losses. All-dielectric metasurfaces, with low optical losses, offer an attractive alternative; however, their functionality is often restricted to reflection-mode operation. This work introduces a hybrid metal–dielectric metasurface designed to operate in transmission mode, specifically tailored for molecular identification in MIR biosensing applications. The metasurface comprises germanium (Ge) on aluminum (Al) cylinders atop a calcium fluoride (CaF_2_) substrate, optimized to exhibits a high transmission efficiency of 80 % at a wavelength of *λ* = 4.2 µm, with a narrow full-width-half-maximum of 0.5 µm. By leveraging the hybridization of modes between the Ge and Al layers, the device enables precise spectral filtering. We demonstrate the potential of this metasurface for molecular identification and biosensing applications through numerical simulations and experimental validation. The straightforward fabrication process further highlights the practicality of this approach, paving the way for miniaturized MIR biosensing platforms.

## 1 Introduction

Mid-infrared (MIR) spectroscopy is a critical tool for sensing applications due to its ability to identify specific molecular absorption bands associated with the fundamental vibrational modes of chemical bonds [1]. This technique enables label-free detection and detailed investigation of molecular structures by leveraging the characteristic absorption bands unique to the MIR spectral range [2]. These features allow for the precise identification of chemical bonds and functional groups within molecules, making MIR spectroscopy highly effective for chemical analysis. Additionally, it is widely employed for characterizing biochemical building blocks such as proteins, lipids, and DNA, owing to its unique chemical specificity [3]. However, the sensitivity of MIR spectroscopy is inherently limited when detecting signals from nanometer-scale samples, biological membranes, or low concentrated samples due to the mismatch between MIR wavelengths and molecular dimensions [3], [4], [5]. Consequently, achieving sufficient signal-to-noise ratios often requires large optical path lengths and, by extension, significant sample volumes or concentrated samples. This limitation restricts the applicability of vibrational spectroscopy in several critical fields, including chemical analysis, biosensing and clinical diagnostics [6].

This limitation can be overcome through light–matter interactions with subwavelength resonators. When the resonance of the resonators is spectrally aligned with the characteristic molecular absorption band, the enhanced coupling between the molecules and the resonator can induce changes in either the resonance frequency or its strength, enabling the identification of molecular structures. This principle, known as surface-enhanced infrared absorption (SEIRA), has been demonstrated using various plasmonic approaches [7], [8]. However, the intrinsic ohmic losses in plasmonic metals lead to significant damping, resulting in low-quality-factor (Q-factor) resonances, which reduce the SEIRA signal and limit the overall performance of this technique [3].

These challenges can be addressed by replacing metals with low-loss, all-dielectric metasurfaces. All-dielectric metasurfaces are composed of high-index dielectric subwavelength resonators, which exhibit significantly lower intrinsic losses compared to their metallic counterparts. In addition to their low-loss properties, all-dielectric metasurfaces support the simultaneous excitation of electric and magnetic Mie resonances of comparable strength, in contrast to plasmonic metasurfaces that are predominantly characterized by electric dipolar resonances [9], [10], [11]. The coupling of both electric and magnetic Mie resonances has facilitated the realization of phenomena that remain largely unexplored in plasmonic metasurfaces, such as Fano resonances [12] and unidirectional scattering [13]. All-dielectric metasurfaces have gained significant attention in MIR spectroscopy due to their versatile applications. For instance, a pixelated dielectric metasurface platform, comprising a series of ultra-sharp resonances each precisely tuned to a specific wavelength, has been employed to detect characteristic molecular absorption at multiple spectral points. This feature allows the conversion of the resulting spectra into a spatial absorption map akin to a barcode [3]. Additionally, all-dielectric metasurfaces have been successfully utilized for highly sensitive biosensing applications [14]. However, these metasurfaces are primarily constrained to reflection mode operation, as they typically generate transmission valleys or notches rather than enabling transmission. Consequently, there is a clear need for an MIR bandpass filtering approach that operates in transmission mode, delivers high transmission efficiency with a narrow FWHM, and provides strong localized field enhancement.

To overcome the limitation of reflection-only operation, transmission-mode devices offer several distinct advantages for MIR applications. Transmission-mode operation enables the design of compact optical systems in which light passes through the filter and reaches the detector directly, eliminating the need for additional optical components such as beam splitters or mirrors to redirect reflected light. This not only reduces system complexity and insertion loss but also minimizes background noise caused by substrate reflections. Furthermore, many real-world MIR systems – including multispectral imaging platforms [15], compact MIR spectrometers [16], and thermal cameras [17] – are inherently designed for transmission-based operation, where the transmitted signal is collected directly by the detector. Transmission-mode filters also offer straightforward integration into collinear optical setups, supporting space-efficient designs and enabling simple system-level alignment.

While reflection-mode metasurfaces provide benefits in certain scenarios, transmission-mode operation is better aligned with the requirements of compact, portable MIR instrumentation and allows straightforward implementation of multi-pass configurations to enhance sensitivity. These considerations collectively motivated the selection of transmission-mode operation for the design and optimization of the presented hybrid metasurface.

Hybrid metal–dielectric metasurfaces have gained significant attention for their potential to reduce the high ohmic absorption losses typically associated with plasmonic metals, while simultaneously enhancing the relatively low field enhancement observed in all-dielectric metasurfaces [18]. Hybrid metasurfaces have found applications in diverse fields, including higher-order harmonic generation [19], high-directivity nanoantennas [20], and fluorescence enhancement [21]. Moreover, hybrid metasurfaces have been employed to achieve high transmission efficiency responses. For example, hybrid metasurfaces have been utilized to generate high-performance structural colors in transmission mode [22], [23]. Additionally, hybrid metasurfaces were demonstrated to generate high transmission responses at an operating wavelength of 2.6 µm for CO_2_ sensing [24].

Nevertheless, a notable challenge remains in the development of metasurfaces operating in transmission mode at longer MIR wavelengths (>4 µm). This challenge arises from the necessity to scale up the unit cell dimensions to accommodate the increased optical path lengths required at these extended wavelengths. As a result, this scaling induces the excitation of multiple resonant modes [25], complicating the design and implementation of hybrid metasurfaces for efficient operation in these higher wavelength regimes.

Here, we present a hybrid metal–dielectric metasurface composed of aluminum (Al) and germanium (Ge) for MIR spectral bandpass filtering applications. The operating wavelength range of the metasurface extends from 3.8 µm to 5.2 µm. We optimized the spectral response of the presented metasurface to achieve the highest transmission at a center wavelength of approximately 4.2 µm. This wavelength lies within an important spectral region in MIR vibrational spectroscopy, overlapping with the strong asymmetric stretching absorption band of CO_2_ near 4.26 µm, which is widely used for gas sensing and atmospheric monitoring. This spectral region is also close to the fundamental C≡N stretching vibration of nitriles (≈4.45 µm), which serve as valuable vibrational probes in studies of proteins, lipids, enzyme interactions, and molecular dynamics [26], [27], [28], [29]. Although our current design is optimized near 4.2 µm, the same design framework can be readily retuned toward the nitrile absorption band, enabling potential applications in nitrile-based sensing and molecular spectroscopy.

The reported metasurface demonstrates a high transmission efficiency of 80 % at a wavelength of 4.2 µm, with a FWHM of 0.5 µm. The metasurface has been comprehensively studied through numerical simulations, fabricated, and experimentally characterized. Detailed multipole expansions reveal that the combination of electric dipole (ED) resonances in the Al metasurface with ED, magnetic dipole (MD), electric quadrupole (EQ), and magnetic quadrupole (MQ) resonances in the Ge metasurface leads to a high transmission efficiency within the hybrid metasurface at 4.2 µm. Furthermore, the spectral response of the hybrid metasurface can be finely tuned across the MIR wavelength range by modifying its geometric parameters, enabling the design of customizable bandpass filters with variable center wavelengths (CWL). A simplified fabrication approach was employed, involving a single lithography step, followed by the deposition of metal and dielectric layers, and a standard lift-off process to define the metasurface features.

## 2 Results

**Figure 1:**
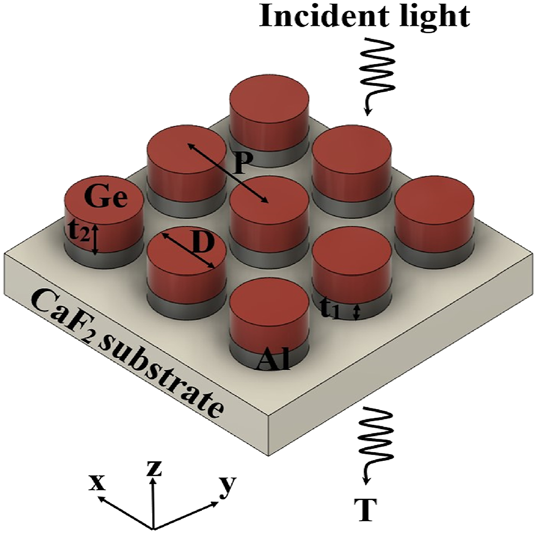
Schematic illustration of the hybrid metal–dielectric metasurface, highlighting all the geometrical parameters.

Figure 1 illustrates the schematic of the hybrid metal–dielectric metasurface, highlighting its geometrical parameters. These parameters include *P*, representing the periodicity (center-to-center distance), *D*, the pillar diameter, and *t*_1_ and *t*_2_, the thicknesses of the gold (Au) and germanium (Ge) layers, respectively. Each unit cell consists of Ge positioned on top of aluminum (Al) pillars, which are placed on a calcium fluoride (CaF_2_) substrate. CaF_2_ was selected for the substrate because of its relatively low refractive index (1.4) and negligible losses in the MIR spectrum [30]. Al was selected as the plasmonic material in this work due to its favorable optical performance in the MIR regime and its compatibility with scalable nanofabrication. Simulations comparing Al with conventional plasmonic metals such as gold (Au) under identical design parameters showed that Al, when paired with Ge, resulted in stronger transmission peaks and narrower resonance linewidths. These findings demonstrate that Al can support high-quality resonances within hybrid metal–dielectric metasurfaces. Beyond this specific implementation, Al is widely regarded as a viable plasmonic material across a broad spectral range – from the ultraviolet to the MIR – and has been effectively employed in advanced nanophotonic applications, including dual-band plasmonic spectroscopy systems [31]. Furthermore, alternative non-noble plasmonic materials, such as indium tin oxide (ITO), have also been successfully demonstrated in MIR biosensing platforms based on surface-enhanced infrared absorption (SEIRA), further underscoring the growing interest in unconventional plasmonic materials for infrared applications [32]. In addition to its optical advantages, Al offers several important practical benefits. It is inexpensive, earth-abundant, and fully compatible with CMOS processing, making it highly suitable for large-scale manufacturing. Al also naturally forms a stable, self-limiting oxide layer, providing environmental resilience without the need for additional surface treatments [18]. Moreover, Al adheres well to CaF_2_ substrates without requiring adhesion-promoting layers, such as chromium or titanium, which are typically needed for Au. These additional layers not only complicate the fabrication process but may also introduce optical losses at the interface. Taken together, these considerations make Al an excellent candidate for plasmonic metasurfaces that demand both high performance and fabrication simplicity. Ge was selected as the dielectric material for its high refractive index and low loss in the MIR region [33]. This design was selected for its fabrication simplicity, low sensitivity to polarization change, high tolerance to variations in incident angles, and the well-known characteristics of the unit cells [34], [35].

The optical response of the hybrid metal–dielectric metasurfaces is driven by the interaction between the plasmonic modes of the metallic component and the Mie resonances of the dielectric layer. This hybridization enables enhanced spectral performance [22]. Comprehensive parametric sweeps of all geometric parameters of the hybrid metasurface were performed to precisely control the spectral position of the transmission response, maximize transmission efficiency, minimize the FWHM, and suppress secondary spectral features at the operating wavelength of *λ* = 4.2 µm. During the parametric sweeps, each parameter was varied independently while keeping the others fixed. All simulations were performed using Lumerical FDTD [36]. In all simulations, an *x*-polarized plane wave was used as the input with periodic boundary conditions applied along the *x* and *y* axes, and perfectly matched layers (PMLs) along the *z*-axis. The mesh size was uniformly set at 5 nm for all simulations. The complex dispersive refractive indices for Al were determined using a multi-coefficient model implemented within Lumerical FDTD, while the refractive indices of thin-film germanium (Ge) were experimentally measured using a Woollam M-2000 ellipsometer.

**Figure 2:**
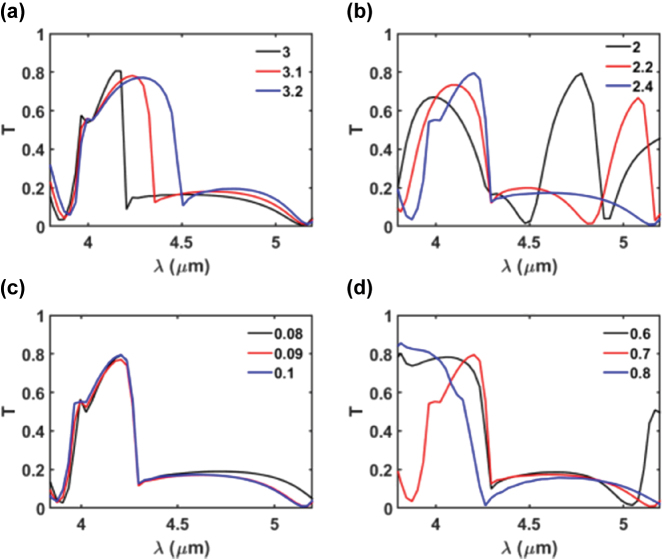
Variation of transmission *T* as a function of wavelength *λ* for different geometric parameters: (a) *P* while keeping *D* = 2.2 µm, *t*_1_ = 0.08 µm, *t*_2_ = 0.6 µm, (b) *D* while keeping *P* = 3.1 µm, *t*_1_ = 0.08 µm, *t*_2_ = 0.6 µm, (c) *t*_1_ while keeping *P* = 3.1 µm, *D* = 2.4 µm, *t*_2_ = 0.6 µm, and (d) *t*_2_ while keeping *P* = 3.1 µm, *D* = 2.4 µm, *t*_1_ = 0.1 µm. The figures illustrate the impact of varying geometrical parameters on the spectral response of the metasurface.

Figure 2(a) depicts the variation in transmission efficiency as a function of *λ* for different periodicities (*P*), while keeping other parameters constant at *D* = 2.2 µm, *t*_1_ = 0.08 µm, and *t*_2_ = 0.6 µm. The periodicity of the unit cells induces proximity resonances between adjacent unit cells. As a result, altering the periodicity (*P*) influences the coupling interactions between neighboring cells, subsequently impacting the transmission efficiency [25]. As *P* increases, the gap between the adjacent pillars widens, reducing the coupling between adjacent pillars and leading to a gradual decrease in transmission efficiency, as shown in Figure 1(b) [22], [25]. Moreover, increasing *P* results in a redshift of the transmission peak and a noticeable narrowing of the FWHM. For example, as *P* increases from 3.0 µm to 3.2 µm, the resonance wavelength shifts from 4.1 µm to 4.3 µm, while the peak transmission slightly decreases from 80.6 % to 78.7 %. Concurrently, the FWHM is reduced significantly from 0.81 µm to 0.39 µm. A period of *P* = 3.1 µm was selected for the final structure, as it offers a desirable trade-off: high transmission efficiency (78.2 %) and a narrow FWHM (0.39 µm) centered around the target wavelength of 4.2 µm. It should be noted that although the spectra at larger periods may visually appear broader, due to reduced peak height and more gradual side slopes – the actual FWHM values, determined through quantitative analysis, clearly exhibit a narrowing trend with increasing period.

Figure 2(b) shows the effect of changing the diameter (*D*) while keeping other parameters fixed at *P* = 3.1 µm, *t*_1_ = 0.08 µm, and *t*_2_ = 0.6 µm. The figure demonstrates that increasing *D* enhances transmission efficiency, induces a redshift in the transmission peak, and suppresses transmission at non-resonant secondary spectral features. The redshift occurs because changing the pillar diameter while keeping the periodicity constant changes the filling factor of the hybrid metasurfaces. According to principles of constructive interference and equivalent refractive index, modifying either the thickness or the filling factor of the metasurfaces affects the effective optical path length, thereby shifting the transmission peaks [37], [38]. Additionally, reducing *D* increases the separation between adjacent unit cells, which diminishes their coupling and leads to enhanced transmission at non-resonant peaks [22]. Figure 2(b) reveals two resonant peaks at *D* = 2 µm: the first at *λ* = 4 µm and the second at *λ* = 4.8 µm. When *D* is increased to 2.2 µm, these spectral peaks are redshifted. For the final design, *D* = 2.4 µm was chosen as it provides a high transmission efficiency (80 %) at *λ* = 4.2 µm and a single resonant peak across the operating wavelength range, thereby minimizing crosstalk.

Figure 2(c) illustrates the influence of changing the thickness of (*t*_1_) on the transmission efficiency while keeping other parameters constant at *P* = 3.1 µm, *D* = 2.4 µm, and *t*_2_ = 0.6 µm. As shown in the figure, the thickness *t*_1_ only slightly alters the transmission efficiency without affecting the spectral position of the transmission peak. A thickness of *t*_1_ = 0.1 μm was selected for optimal transmission efficiency (80 %) at *λ* = 4.2 μm and FWHM of 0.5 µm. In contrast, the parameter *t*_2_ exerts a more pronounced influence on the spectral response, as verified through extended parametric sweeps. Even when swept across a broader range, *t*_1_ consistently exhibited limited impact on performance. Therefore, *t*_1_ was kept minimal to reduce deposition time and facilitate fabrication. As illustrated in Figure 2(d), increasing *t*_2_ results in a redshift of the transmission peak. This redshift is attributed to the increased effective optical path length associated with the greater thickness [38]. A thickness of *t*_2_ = 0.7 μm was chosen as it provides the optimal transmission performance, balancing both transmission efficiency and the FWHM of the transmission peak, as depicted in Figure 2(d). Following the parametric sweeps, the optimized geometrical parameters of the design were determined as *P* = 3.1 µm, *D* = 2.4 µm, *t*_1_ = 0.1 µm, and *t*_2_ = 0.7 µm. These values yielded a transmission efficiency of approximately 80 % at the target wavelength of *λ* = 4.2 μm and FWHM of 0.5 µm.

To emphasize the benefits of hybrid metal–dielectric metasurfaces and their contributions to the overall response, we computed the transmission spectra of the Al only, Ge only, and hybrid Al–Ge metasurfaces, using the optimal geometrical parameters. Additionally, we performed multipole expansions and calculated the scattering cross section (SCS) for both the single and the hybrid metasurfaces, to elucidate the underlying mechanisms behind the hybrid metasurface response.

An open-source MATLAB code, multipole expansion for nanophotonics (MENP) [39], was utilized to simulate both the multipole expansion and the scattering cross section (SCS). MENP is a MATLAB-based program used to compute electric dipole (ED), magnetic dipole (MD), electric quadrupole (EQ), and magnetic quadrupole (MQ), followed by the SCS calculation. Initially, the electric fields were generated using Lumerical FDTD simulations and subsequently stored in .mat files, along with the corresponding refractive index distribution (*n*(*x, y, z, f*)) and one-dimensional axis arrays (*x, y, z, f*). These data were then processed by MENP, where the electric field distributions were first transformed into current density. This current density was used to compute the four multipolar modes (ED, MD, EQ, and MQ) and then the SCS.

**Figure 3:**
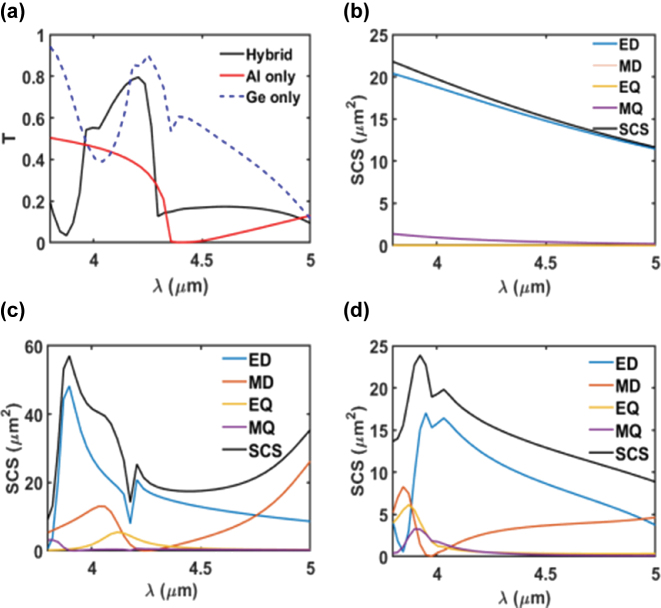
Transmission and multipole analysis of the metasurfaces. (a) Transmission (*T*) as a function of wavelength (*λ*) for the Al only, the Ge only, and the hybrid metasurfaces, highlighting the effect of using hybrid metasurface on the spectral response of the metasurface. The multipole expansions of the SCS in terms of ED, MD, EQ, and MQ contributions are shown for (b) the Al only, (c) the Ge only, and (d) the hybrid metasurface.

Figure 3(a) illustrates the transmission spectra of the Al only, Ge only, and hybrid metasurfaces at the optimized geometrical parameters. The results indicate that the Al only metasurface exhibits a low transmission of approximately 30 % at *λ* = 4.2 µm. In contrast, the Ge only metasurface shows a high transmission across the operating wavelength range. The absence of sharp spectral features in both the Al only and Ge only metasurfaces, as observed in the figure, is disadvantageous for sensing applications, which typically require one or two well-defined peaks within the operating range [40]. The figure depicts that the addition of Ge to the Al metasurface significantly enhances the transmission at the target wavelength of 4.2 µm while effectively suppressing transmission at other wavelengths.

Next, we performed calculations of the multipole expansions and the SCS for the single and hybrid metasurfaces to understand the physical origin of the resonant mode and to determine the contributions of the Al and Ge metasurfaces to the resonant response of the hybrid metasurface. Figure 3(b)–(d) show the multipole expansions and the SCS of the Al only, the Ge only, and the hybrid metasurfaces, respectively.

Figure 3(b) illustrates that the Al only metasurface exhibits a prominent and broad ED resonance. Due to the inherently non-directional nature of electric dipolar scattering [41], the Al-only metasurface shows low transmission without any sharp spectral features, as shown in Figure 3(a).

Figure 3(c) demonstrates that the Ge only metasurface produces a relatively minor contribution from higher-order resonant modes, such as the MQ and the EQ, in addition to the dominant ED and MD modes. This observation aligns with previous studies suggesting that dielectric metasurfaces excite higher-order modes [40]. The hybridization of Al and Ge results in enhanced transmission at *λ* = 4.2 μm, attributed to the excitation of ED, MD, EQ, and MQ modes within the hybrid metasurface. However, Figure 3(d) indicates that the spectral response of the hybrid metasurface is shifted away from *λ* = 4.2 μm. This shift is likely due to approximations involved in the calculations of the SCS and multipole expansions [39].

**Figure 4:**
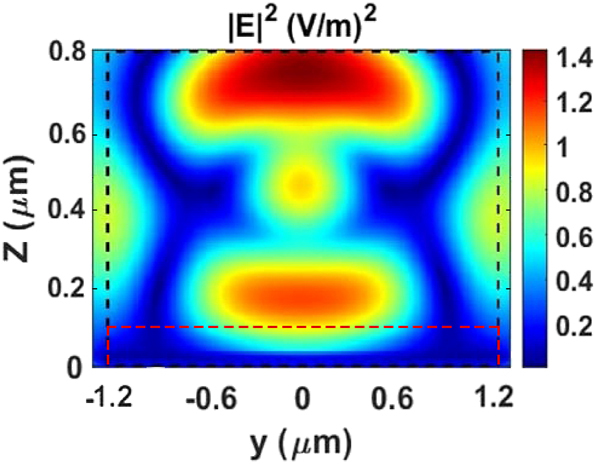
The electric field distribution of the hybrid metasurface at a wavelength of *λ* = 2.6 µm, as observed in the *yz* plane. The red and black dashed lines indicate the positions and boundaries of the Ge and Al cylindrical structures, respectively.

Figure 4 depicts the electric field (E-field) distribution of the hybrid metasurface in the *yz* plane. The figure reveals that the E-field is predominantly concentrated around the unit cells of the metasurface, indicating a high sensitivity to the surrounding environment which is useful for refractive index sensing applications [18]. The red and black dashed lines in the figure indicate the positions of the Ge and Al cylindrical components, respectively.

## 3 Experiments

Hybrid metasurfaces are typically fabricated using either a single lithography step [18] or two lithography steps [42]. The latter increases fabrication time and cost and requires precise alignment between successive patterning steps, which can be particularly challenging for high-resolution features. While single-step lithography offers a more streamlined approach, it typically necessitates reactive ion etching (RIE), which poses difficulties in achieving highly anisotropic etching profiles, especially for thicker metasurface structures. In this work, we adopt a simplified fabrication strategy that employs a single lithography step combined with a standard lift-off process. This approach eliminates the need for etching or additional lithographic alignment, thereby reducing process complexity while maintaining the required structural fidelity.

**Figure 5:**
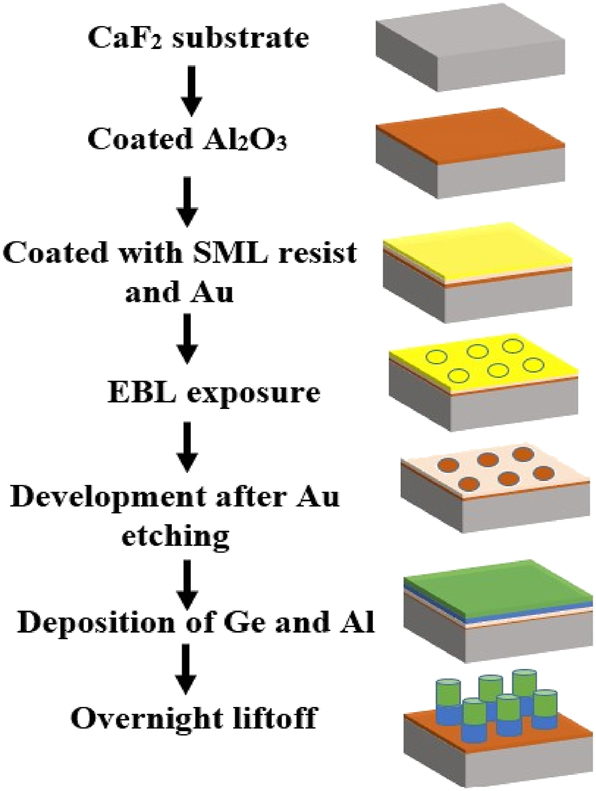
A schematic illustrating the fabrication process for the hybrid metal–dielectric metasurface. The process begins with a CaF_2_ substrate, followed by Al_2_O_3_ deposition, coating with SML resist and a thin Au layer, EBL exposure, and development. After Au etching, Ge and Al are deposited via thermal evaporation. The process ends with an overnight lift-off step to define the final metasurface structures.

**Figure 6:**
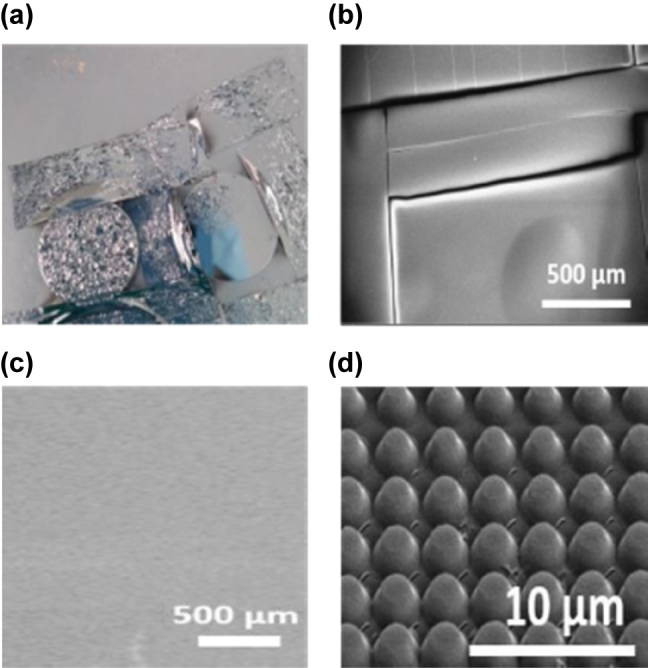
Deposition of Ge on CaF_2_ substrate and SEM of the fabricated metasurface (a) Ge film deposited on a CaF_2_ substrate without an adhesion layer. (b, c) SEM images of Ge deposited on CaF_2_ substrates using different deposition rates: (b) 4 Å/s and (c) 1 Å/s. (d) Tilted SEM image of the fabricated metasurface, showing a truncated-cone profile.

The fabrication process, summarized in the schematic in Figure 5, begins with the cleaning of a CaF_2_ substrate using acetone, followed by isopropyl alcohol (IPA) to remove any residual organic contaminants. Initially, Ge was deposited directly onto the substrate; however, adhesion issues were observed, as shown in Figure 6(a). Metal oxides, such as magnesium oxide (MgO), are commonly used as adhesion layers for CaF_2_ substrates [33]. Hence, an adhesion layer of aluminium oxide (Al_2_O_3_) was introduced. Al_2_O_3_ was selected for its strong adhesive properties, which prevent the Ge layer from detaching while maintaining the optical performance of the metasurface. Numerical simulations confirmed that the Al_2_O_3_ layer had no adverse effects on the optical response of the metasurface. A 15 nm Al_2_O_3_ layer was deposited via atomic layer deposition (Fiji, Veeco) to optimize the adhesion between the Ge and CaF_2_ substrates without influencing the optical response of the metasurface.

Next, SML electron beam resist (EM Resist Ltd.) was spin-coated onto the substrate for electron beam lithography (EBL) patterning. The resist was soft-baked at 160 °C for 4 min, a temperature that successfully eliminated cracks in the resist film, as observed previously [24]. The use of SML enabled a simplified fabrication process, requiring only a single EBL step instead of a two-step procedure.

Then, a 15 nm Au layer was deposited onto the resist to dissipate excess charges during the EBL exposure process. A dose test was performed to determine the optimal dose for achieving accurate dimensions, with a range from 1,250 to 1,310 μC/m^2^, with 20 μC/m^2^ increments. Following EBL exposure, the Au layer was removed using a gold etchant supplied by Sigma-Aldrich (30 s in standard gold etchant from Sigma-Aldrich). The resist was then developed in MIBK: IPA (1:3) for 15 s, followed by immersion in IPA for another 15 s to stop development.

After EBL, Al and Ge were deposited using a thermal evaporator (MiniLab 60, Moorfield Nanotechnology Ltd). Initially, Ge was deposited at a rate of 4 Å/s. SEM image of the films deposited at a rate of 4 Å/s, shown in Figure 6(b) reveals the presence of cracks in the deposited film. High deposition rates have been reported to induce compressive stresses within the deposited film, leading to the formation of cracks [43], [44]. Conversely, lower deposition rates reduce the induced stresses but require extended deposition times, particularly for thicker films. To mitigate the crack formation, the deposition rate was subsequently reduced to 1 Å/s. As depicted in Figure 6(c), the Ge film deposited at 1 Å/s exhibited no cracks, confirming that the lower deposition rate effectively minimized stress and prevented crack formation. Therefore, a deposition rate of 1 Å/s was employed for all subsequent depositions. After the Al and Ge deposition, the substrates were immersed in acetone overnight for the lift-off process. Figure 6(d) presents an SEM image of the fabricated hybrid metasurface using an EBL dose of 1,250 μC/m^2^. The image reveals some shape deformation, as the fabricated metasurface pillars exhibit a frustum (truncated cone) shape rather than uniform cylindrical structures. This deformation occurs during the deposition of thicker films, where the deposited layer tapers as it thickens. This effect results from the accumulation of material on top of the resist, leading to a reduction in feature size as the layer increases in thickness.

## 4 Characterization

The optical characterization of the fabricated hybrid metal–dielectric metasurfaces was conducted using a Fourier transform infrared (FTIR) microscope (Shimadzu AIM-9000). All the measurements were performed in transmission mode under 0° light incidence. A bare CaF_2_ substrate was initially measured as the reference for the subsequent measurements.

**Figure 7:**
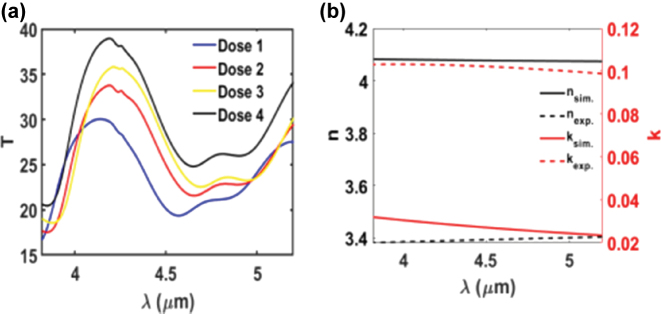
FTIR spectra of metasurfaces and optical constants of germanium. (a) FTIR measurements of various metasurfaces fabricated with different EBL doses, where each measurement corresponds to a distinct metasurface. (b) Simulated and measured refractive index of germanium (Ge), showing both the real (*n*) and imaginary (*k*) parts.

Four metasurface samples were fabricated using different electron beam lithography (EBL) doses – 1,250, 1,270, 1,290, and 1,310 µC/cm^2^ – and their transmission spectra were systematically characterized, as shown in Figure 7(a). The applied dose serves as a reproducible proxy for controlling the microdisk diameter, with higher doses generally resulting in larger features under consistent fabrication conditions. This approach has been widely adopted in dielectric metasurface fabrication for dose-based structural tuning [11]. The transmission spectra reveal a clear spectral shift in response to dose variation. As the EBL dose increases from 1,250 to 1,290 μC/cm^2^, a redshift in the resonance wavelength is observed, attributed to the increase in disk diameter, which leads to a larger effective mode volume and consequently a lower resonant frequency [11]. At the highest dose (1,310 μC/cm^2^), a slight blueshift is detected, which is attributed to the narrowing of inter-disk gaps that enhances near-field coupling between adjacent resonators. This coupling effect reduces the effective refractive index of the resonant mode, thereby shifting the resonance to shorter wavelengths. Similar coupling-induced blueshifts have been reported in dielectric dimers [11] and silicon nanodisk arrays [45]. These results confirm that the resonance wavelength of the metasurface can be finely tuned across the MIR spectrum by controlling the structural dimensions through dose modulation. When comparing these measurements with the simulation results depicted in Figure 3(a), it is evident that both exhibit the same spectral position. However, the measured results show reduced transmission efficiency, and a lower Q-factor (broader response) compared to the simulations. This decrease in transmission efficiency is attributed to the surface roughness of the Al–Ge pillars, which resulted from the shape deformation observed in Figure 6(d). The surface roughness introduces additional scattering of the incident light, thereby diminishing the measured transmitted light [25]. Moreover, it has been reported that imperfections in fabrication and surface roughness of the metasurface unit cells contribute to the broadening of the resonance [46], [47]. Consequently, the surface roughness of the metasurface unit cells leads to increased light scattering, which simultaneously results in lower transmission efficiency and a broader response. The surface roughness of the deposited Ge can be substantially minimized by employing alternative deposition techniques such as E-beam evaporation or sputtering.

The broadened spectral widths also indicate significant light leakage and reduced field confinement. The extent of optical confinement in a metasurface response is primarily determined by the refractive index contrast between the metasurface and its surrounding environment [48]. A higher refractive index contrast results in stronger photon confinement within the metasurface. The metasurfaces are typically exposed to air, hence the refractive index of the metasurface’s material becomes the key factor in determining the degree of field confinement and consequently affecting the spectral width of the resonance. Metasurfaces with a higher refractive index exhibit a greater refractive index contrast, thereby confining light more effectively than those with a lower refractive index. To assess whether the fabricated metasurfaces matched the simulated parameters, the optical constants of the deposited Ge were measured to confirm matching with the high refractive index used in the simulations.

The optical constants of the deposited Ge were determined through spectroscopic ellipsometry measurements using a Woollam Ellipsometer M-2000. Data were collected at incidence angles of 45°, 50°, 55°, and 60°. The measured spectra were fitted to the calculated spectra using a multilayer model, with the refractive index extracted through modeling based on the Genosc model [49]. The fitted spectra closely matched the measured data across all wavelengths and angles, allowing for the calculation of the optical constants of the deposited Ge, as shown in Figure 7(b).

Figure 7(b) presents a comparison between the refractive index of the simulated Ge and the experimentally measured Ge. The results indicate that the measured refractive index exhibits a lower real component and a higher imaginary component than the simulated values. This discrepancy suggests that the optical properties of the deposited Ge deviate from those used in the simulations. The reduced real part of the refractive index lowers the refractive index contrast between the metasurfaces and their surrounding environment, thereby diminishing field confinement and broadening the spectral response relative to the simulated results [48]. In addition, the increased imaginary component corresponds to higher optical absorption, which contributes to the reduced transmission efficiency observed in the experimental measurements compared to the simulations.

The refractive index used in the simulations was selected from the Lumerical FDTD material database, which includes widely adopted dispersive models that closely reflect values reported in the literature for germanium. This model was chosen during the initial design phase to ensure consistency with established theoretical frameworks and to facilitate comparison with prior work. Although the refractive index of the deposited Ge film was later measured experimentally, the original model was retained throughout the simulation process to maintain consistency across the design workflow and to avoid reparameterizing the full simulation space. The deviations between the simulated and measured optical constants – particularly in terms of real part reduction and increased loss – largely account for the discrepancies observed between the simulated and experimental transmission spectra.

In addition to the effects of surface roughness and material property deviations discussed above, the remaining discrepancies between the simulated and experimental spectra can be attributed to several contributing factors. Fabrication tolerances primarily arise from the electron-beam lithography process, where slight variations in local dose distribution, proximity effects, and resist development can result in nanoscale deviations of pillar diameter and inter-pillar spacing. Since all devices were fabricated on the same substrate under identical processing conditions, these variations are localized rather than wafer-to-wafer. Such nanoscale differences directly affect the resonance wavelength and spectral width. Surface roughness and sidewall profile variations introduce additional scattering losses, lowering transmission and broadening the response, as observed in Figure 6(d). Material property variations also contribute: the experimentally measured Ge film exhibited a lower real refractive index and higher extinction coefficient compared to the simulated model (Figure 7(b)), reducing refractive index contrast and increasing absorption loss. Finally, measurement conditions, including slight defocus in the FTIR setup or spatial non-uniformity across the measurement aperture, can lead to underestimation of peak transmission and apparent Q-factor broadening. Taken together, these factors explain the discrepancy between simulated and measured spectra and highlight the importance of dose optimization, resist process control, and material quality for improving performance in future iterations.

To clarify the distinctive advantages of our approach over existing MIR filtering technologies, we compared the performance of the proposed hybrid metasurface filter with representative solutions reported in the literature, including linear variable optical filters (LVOFs), Fabry–Pérot (FP) filters, plasmonic metasurfaces, and other hybrid metal–dielectric metasurfaces [18], [50], [51], [52]. Alternative technologies such as LVOFs typically exhibit transmission efficiencies below 20 %, broad spectral responses, and increased cross-talk resulting from the excitation of multiple spectral features [50]. Similarly, FP filters face challenges including broad spectra and high cross-talk, due to the limited refractive index contrast of MIR-transparent materials [51], and they often require time-intensive multilayer deposition processes with strict thickness tolerances. Plasmonic metasurfaces in the MIR range also suffer from low transmission efficiencies (<40 %) and broad spectral features, primarily due to ohmic losses in metallic components [52]. Previously reported hybrid metal–dielectric metasurfaces for refractive index sensing show multiple overlapping resonances and broad spectral responses, which can limit selectivity and reduce spectral resolution [18].

In contrast, the proposed hybrid metasurface demonstrates a unique combination of high peak transmission (∼80 %), narrow FWHM (∼0.5 µm), and a single, well-isolated resonance that minimizes cross-talk. Moreover, it employs a straightforward, single-step lithographic process, offering a scalable and efficient route for mass fabrication. These combined features position the device as a highly competitive and practical solution for MIR gas sensing, molecular fingerprinting, and spectroscopic applications, where high transmission, narrow spectral response, and fabrication simplicity are critical.

## 5 Application potential and spectral tunability

While this work focused on validating the optical response of the hybrid metasurface, the presented design can be directly used for MIR applications such as CO_2_ detection, as its passband is centered near 4.2 µm where CO_2_ exhibits strong absorption. Furthermore, the metasurface can be readily retuned toward ≈ 4.45 µm by adjusting the dielectric thickness (*t*_2_) and pillar diameter (*D*), enabling measurements of nitrile-containing thin films or flow-cell samples. This spectral tunability highlights the versatility of the proposed approach for gas sensing, molecular fingerprinting, and biosensing, where narrowband, high-transmission filters are crucial for selectively probing characteristic vibrational modes of target analytes. The demonstrated design therefore establishes a solid foundation for future integration into compact MIR spectroscopic and biosensing platforms.

## 6 Conclusion

In conclusion, we have demonstrated the successful realization of a hybrid metal–dielectric metasurface, comprising Ge on Al cylinders. The metasurface shows significant promise as a high-performance MIR optical filter operating in transmission mode. Specifically, a bandpass filter targeting 4.2 µm was designed, achieving a transmission efficiency of 80 % with FWHM of 0.5 µm. The optical properties of the metasurface were rigorously examined through numerical simulations, and the operating principles of the hybrid metal–dielectric structure were further elucidated using multipole expansions. The optical response of the metasurface can be tuned across the MIR spectrum by adjusting its geometrical parameters, offering versatile functionality for various applications, including molecular spectroscopy and biosensing. Furthermore, we successfully implemented a straightforward, single-step lithographic nanofabrication process to fabricate the metasurface. This fabrication method provides a scalable approach for more complex metasurfaces, thus expanding the potential for miniaturized MIR sensing platforms.
